# Risk factors associated with neonatal mortality among neonates admitted to neonatal intensive care unit of the University Teaching Hospital in Lusaka

**DOI:** 10.1038/s41598-024-56020-6

**Published:** 2024-03-04

**Authors:** Deborah Tembo, Francis D. N. Abobo, Patrick Kaonga, Choolwe Jacobs, Barnabas Bessing

**Affiliations:** 1 School of Public Health, Department of Epidemiology and Biostatistics, University of Zambia, Lusaka, Zambia; 2 Zambia National Public Health Institute, Lusaka, Zambia; 3 World Health Organization Country Office, Maseru, Lesotho; 4 World Health Organization Country Office, Lusaka, Zambia

**Keywords:** Neonates mortality, NICU, Risks factors, Trends, Zambia, Health care, Medical research, Risk factors, Signs and symptoms

## Abstract

Globally, several children die shortly after birth and many more of them within the first 28 days of life. Sub-Sharan Africa accounts for almost half (43%) of the global neonatal death with slow progress in reduction. These neonatal deaths are associated with lack of quality care at or immediately after birth and in the first 28 days of life. This study aimed to determine the trends and risk factors of facility-based neonatal mortality in a major referral hospital in Lusaka, Zambia. We conducted retrospective analysis involving all neonates admitted in the University Teaching Hospital Neonatal Intensive Care Unit (UTH-NICU) in Lusaka from January 2018 to December 2019 (N = 2340). We determined the trends and assessed the factors associated with facility-based neonatal mortality using Generalized Linear Models (GLM) with a Poisson distribution and log link function. Overall, the facility-based neonatal mortality was 40.2% (95% CI 38.0–42.0) per 1000 live births for the 2-year period with a slight decline in mortality rate from 42.9% (95% CI 40.0–46.0) in 2018 to 37.3% (95% CI 35.0–40.0) in 2019. In a final multivariable model, home delivery (ARR: 1.70, 95% CI 1.46–1.96), preterm birth (ARR: 1.59, 95% CI 1.36–1.85), congenital anomalies (ARR: 1.59, 95% CI 1.34–1.88), low birthweight (ARR: 1.57, 95% CI 1.37–1.79), and health centre delivery (ARR: 1.48, 95% CI 1.25–1.75) were independently associated with increase in facility-based neonatal mortality. Conversely, hypothermia (ARR: 0.36, 95% CI 0.22–0.60), antenatal attendance (ARR: 0.76, 95% CI 0.68–0.85), and 1-day increase in neonatal age (ARR: 0.96, 95% CI 0.95–0.97) were independently associated with reduction in facility-based neonatal mortality. In this hospital-based study, neonatal mortality was high compared to the national and global targets. The improvement in neonatal survival observed in this study may be due to interventions including Kangaroo mother care already being implemented. Early identification and interventions to reduce the impact of risks factors of neonatal mortality in Zambia are important.

## Introduction

Neonatal mortality is the death of newborns during the first 28 days of life after a live birth. Globally, 47% of the mortality in children under-5 years are attributed to neonatal death^1,2^. The burden of neonatal mortality continues to be a public health concern, especially in developing countries^3^. For instance, in 2019, 2.4 million neonates died globally with sub-Saharan Africa accounting for 41% of the neonatal death^1,4^. The risk of neonatal mortality is thirty times higher in sub-Saharan Africa compared with the lowest neonatal mortality in the United States^2^. Even though neonatal mortality estimates in sub-Saharan Africa have reduced from 45.6 deaths per 1000 livebirths in 1990 to 27.1 deaths per 1000 livebirths in 2019, this mortality risk is still unacceptably high^5,6^.

Several factors including sociodemographic, reproductive health, perinatal care, child-feeding practices are found to be associated with increases in general population and facility-based neonatal mortality rates especially in developing countries. For instance, lower maternal educational levels^7,8^, place of delivery and residence^3,9,10^, maternal age^4,7,9,11^, parity^11^, gestational age^4,7,9^, inadequate antenatal visits^12,13^, newborn sex^3^, newborn age^14,15^, mode of delivery^16,17^, low birthweight^10,15^, both maternal and foetal complications^14^, preterm birth^15^, low Apgar score^18^, perinatal asphyxia^19^, congenital anomalies^14^, neonatal infections^20^, hypothermia^21^, respiratory distress syndrome^20^, late initiation and or lack of exclusive breastfeeding^22^ are significantly associated with neonatal mortality in developing countries. Interventions addressing issues during pregnancy, intrapartum care, nutrition, and postnatal care including proper care for small and sick newborns can significantly reduce the risk of both facility-based and general neonatal mortality.

In Zambia, neonatal mortality decreased from 37 in 2001 to 27 deaths per 1000 live births in 2018^23^. Several strategies, policy interventions and program implementations contributed to this progress in the neonatal mortality reduction. Programs such as helping baby breath campaign, Emergency Obstetric and Neonatal Care (EmONC) training and implementation, Saving Mothers Giving Life (SMGL), Safe Motherhood 360 + projects, decentralization of mother and newborn care, amongst others are being implemented to reduce neonatal mortality to 12 per 1000 live births by 2030 according to the Sustainable Development Goals (SDG)^3,10,19^.

It is important to highlight the pattern of neonatal mortality and associated risk factors especially in referral health facilities, adding to the limited scientific evidence on the progress made and challenges. This may contribute to policy-directions in Zambia on targeted interventions towards attaining its’ neonatal mortality reduction goal by 2030. In this study, we aimed to estimate the neonatal mortality rates and investigate the risk factors associated with neonatal mortality in a major referral health facility for targeted interventions.

## Methods

### Study design and settings

This is a retrospective descriptive study using neonatal admissions records (January 1, 2018–December 31, 2019) of the Neonatal Intensive Care Unit (NICU) at the women and new-born hospital of the University Teaching Hospital (UTH), Zambia. UTH is the largest tertiary hospital which trains all calibre of health professionals and serves as a major referral hospital in Zambia. It is located approximately four kilometres east of the city centre of the capital city of Lusaka.

### Study participants, sampling procure and sample size

In this study, a neonate is defined as any newborn less than 28 days of life. The study population included all neonates admitted to the NICU with complete information on required variables in the first 28 days of life at the NICU either discharge alive/death or still on admission at 28 days maximum. A complete enumeration method was used and all reported neonates admitted to the NICU over the study period were sampled for data completeness and inclusion. The data was extracted from the neonatal case records of the UTH-NICU health management information systems. Hard copies of individual case files were collected from the medical records and required information extracted into predesigned excel-based data collection tool with variables of interest. The data was collected by the primary author and validated by two co-authors for completeness and accuracy. Data cleaning, coding and cross-validation with the original data source was done by the researchers before it was exported into STATA version BE/18.0 for analyses.

**Figure 1:**
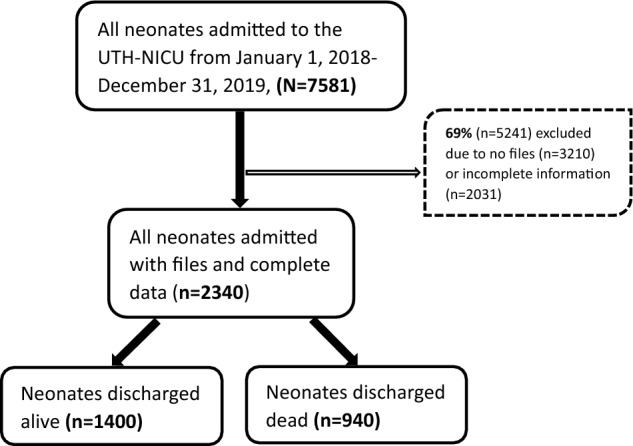
Study participant sampling flow chart.

A total of 7581 neonatal admissions were recorded during the study period. Of these, more than half (69%) were excluded (3210 missing files and 2031 incomplete data) and 31% (2340) consisting of 1197 in 2018 and 1143 in 2019 were included in the final analyses (see Fig. 1). Assuming a minimum power of 80% using the freedom method^24^, the included records were sufficient enough to detect at least 8% difference in our facility-based neonatal mortality compared with the general neonatal population mortality in Zambia with 95% precision and 5% significance level^25^.

### Measures

The dependent (outcome) variable was neonatal mortality among neonates admitted at the NICU which was measured as a binary outcome: died or alive at the time of discharge from the NICU or 28 days maximum. If a woman had a live birth but lost the neonate within the first 28 days after birth it was coded as “1” or otherwise coded “0”.

The independent variables included sociodemographic, maternal, and neonatal related factors. The sociodemographic data that were captured included age of mother (years), age of neonate (days), sex of neonate, and residence. Self-reported maternal related variables captured were parity, gravida, place of birth (hospital, health centre, and home), antenatal attendance (no, yes, and unknown), mode of delivery (spontaneous vaginal, caesarean section, breech-assisted, and vacuum-assisted), category of staff who conducted the delivery (midwife, doctor and unskilled staff), HIV status of mother (known negative, known positive, and unknown). Neonatal related factors included were primary diagnosis on admission to the NICU (birth asphyxia, congenital anomalies, prematurity, respiratory distress syndrome, sepsis, hypothermia, jaundice, glycaemia, stable neonate, and other diagnosis), gestational age (weeks), and birth weight (grams). The gestational age was categorized into preterm (< 37 weeks gestation) and term (≥ 37 weeks gestation). Birth weight was also categorized into low birth weight (< 2500 g) and normal birth weight (≥ 2500 g).

### Statistical analysis

We presented descriptive statistics as medians and interquartile ranges (IQR) for non-normally distributed continuous variables, and frequencies with percentages for categorical variables. The Chi-square test was used for categorical variables and Mann Whitney test for continuous variables to determine if there is significant difference in outcomes amongst the variables. To evaluate associations with neonatal mortality, we used Poisson Regression Models to obtain incident rate ratio (IRR) along with 95% confidence Intervals (CI). We used Generalized Linear Models (GLM) with a Poisson distribution and log link function. This is a flexible model within the GLM that assumes a linear relationship between the independent variables and binary outcome allowing logarithmic transformation of variables to return an exposure coefficient whose natural exponents can be interpreted as risk ratios^26^. We applied robust variance estimator to obtain valid standard errors which accounts for model misspecification that occurs because the binary outcome does not follow a Poisson distribution^26,27^. The model goodness of fit was assessed based on low Akaike Information Criterion (AIC), and likelihood-ratio test.

To identify the factors that remained independently associated with our outcome, we fitted a single multivariable Poisson regression model that, at the outset, included all covariates and potential confounders for which a Wald test of their estimated coefficient yielded *p* < 0.05 in the univariable analyses. This resulted in estimation of adjusted incidence rate ratio (AIRR) with 95% CI. To test the robustness of this method, the model was repeated using automated stepwise backward elimination procedures based on AIC. Both methods yielded the same results after adjusting for time in each model. We also stratified our analyses by gestation (preterm: < 37 weeks, term: ≥ 37 weeks) and birth weight (low birth weight: < 2500 g, normal birth weight: ≥ 2500 g) categories to determine whether these outcomes impact neonatal mortality incidence rate ratios differently. Categorical independent variables (e.g., sex, place of birth, mode of delivery, HIV status, and antenatal attendance) were modelled as dummy variables compared to a reference category and continuous variables as linear predictors after confirming that a linear predictor fitted well. A two-tailed (*p* < 0.05) was considered statistically significant. STATA version BE/18.0 was used to perform all analyses.

### Ethical approval and consent to participate

The University of Zambia Biomedical Research Ethics Committee (UNZBREC) approved the current study and conforms to the 1964 Declaration of Helsinki Ethical Standards for Human Research. The informed consent was waived by UNZBREC due to the retrospective nature of the study.

## Results

### Sample representativeness

We compared participants included (n = 2340) and those excluded that have files but incomplete information (n = 2031) and found no significant differences by gestational age in weeks (*p* = 0.25), neonatal age on admission in days (*p* = 0.42), sex (*p* = 0.71), place of birth (*p* = 0.32), and birthweight (*p* = 0.19).

### Participant characteristics

The participant characteristics shown in Table 1 indicate that the median maternal age was 25 years (IQR: 21–31), median gravida was 2 (IQR: 1–4), median parity was 2 (IQR: 1–3) and median gestation was 36 weeks (IQR: 30–37). The majority (76%) of the mothers were known to be HIV negative, and 84% of them attended antenatal care during their pregnancy.

**Table 1:** Maternal and neonatal characteristics.

Variable	Total neonates (n = 2340)	Neonatal vital status		*p*-value
		Alive (n = 1400)	Died (n = 940)	
Age of mother in years, median (IQR)	25 (21–31)	25 (21–30)	25 (21–32)	0.087
Gravida, median (IQR)	2 (1–4)	2 (1–3)	2 (1–4)	0.425
Parity, median (IQR)	2 (1–3)	2 (1–2)	2 (1–3)	**0.042**
Gestational age in weeks, median (IQR)	36 (30–37)	36 (33–38)	32 (28–36)	** < 0.001**
Age of neonate in days, median (IQR)	4 (2–7)	5 (3–8)	2 (2–5)	** < 0.001**
Sex, n (%)				
Male	1223 (52.3)	758 (54.1)	465 (49.5)	**0.026**
Female	1117 (47.7)	642 (45.9)	475 (50.5)	
Place of birth, n (%)				
Hospital	2131 (91.1)	1319 (94.2)	812 (86.4)	** < 0.001**
Health centre	104 (4.4)	46 (3.3)	58 (6.2)	
Home	105 (4.5)	35 (2.5)	70 (7.5)	
Type of birth, n (%)				
Single	2235 (95.5)	1366 (97.6)	869 (92.5)	** < 0.001**
Twin	105 (4.5)	34 (2.4)	71 (7.6)	
Mode of delivery, n (%)				
Spontaneous vaginal delivery	1937 (82.8)	1228 (87.7)	709 (75.4)	** < 0.001**
Caesarean section	341 (14.6)	166 (11.9)	175 (18.6)	
Assisted breech delivery	49 (2.1)	5 (0.4)	44 (4.7)	
Vacuum-assisted vaginal delivery	13 (0.6)	1 (0.1)	12 (1.3)	
Delivered by				
Midwife	1879 (80.3)	1197 (85.5)	682 (72.6)	** < 0.001**
Doctor	357 (15.3)	168 (12.0)	189 (20.1)	
Unskilled staff	104 (4.4)	35 (2.5)	69 (7.3)	
Maternal HIV status, n (%)				
Known negative	1784 (76.2)	1099 (78.5)	685 (72.9)	**0.004**
Known positive	387 (16.5)	204 (14.6)	183 (19.5)	
Unknown	169 (7.2)	97 (6.9)	72 (7.7)	
Attended antenatal care? n (%)				
Yes	1966 (84.0)	1198 (85.6)	768 (81.7)	** < 0.001**
No	220 (9.4)	79 (5.6)	141 (15.0)	
Unknown	154 (6.6)	123 (8.8)	31 (3.3)	
Preterm birth, n (%)				
Yes (< 37 weeks)	1612 (68.9)	819 (58.5)	793 (84.4)	** < 0.001**
No (≥ 37 weeks)	728 (31.1)	581 (41.5)	147 (15.6)	
Low birth weight, n (%)				
Yes (< 2500 g)	1192 (50.9)	519 (37.1)	673 (71.6)	** < 0.001**
No (≥ 2500 g)	1148 (49.1)	881 (62.9)	267 (28.4)	

*IQR* Interquartile range, *HIV* human immunodeficiency virus.

Significant values are in bold.

Of the 2340 neonates admitted at the NICU during the study period, the median neonatal age at admission was 4 days (IQR: 2–7) with 52% of them being males, 91% were born at the hospitals, 83% were born via spontaneous vaginal delivery, 80% were delivered by a midwife, 69% were born preterm (< 37 weeks gestation), and 51% were low birth weight (< 2500 g). Most of the neonates (60%) were discharged alive and 40% (940) were discharged as died. Compared to those neonates discharged alive, those neonates who were discharged as died were more likely to; be females (51% vs. 46%, *p* < 0.001), have lower gestational age at birth (32 weeks vs. 36 weeks, *p* < 0.001), have short life after birth (2 days vs. 5 days, *p* < 0.001), be born preterm (< 37 weeks vs. ≥ 37 weeks, *p* < 0.001) and have low birth weight (< 2500 g vs. ≥ 2500 g, *p* < 0.001).

### Trends of, and factors associated with, neonatal mortality

In terms of trend, the overall neonatal mortality rate ratio (RR) was 402 (95% CI 380–420, *p* < 0.001) per 1 000 live births for the 2-year period with a slight decline from 429 (95% CI 400–460, *p* < 0.001) in 2018 to 373 (95% CI 350–400, *p* < 0.001) per 1000 live births in 2019 (Table 2).

**Table 2:** Crude neonatal mortality rates.

Year	Number of admissions	Neonatal vital status		Neonatal mortality rate* (95% CI)
		Alive, n (%)	Died, n (%)	
2018	1197	683 (57.1)	514 (42.9)	**429 (400–460)**
2019	1143	717 (62.7)	426 (37.3)	**373 (350–400)**
Total	2340	1400 (59.8)	940 (40.2)	**402 (380–420)**

*Neonatal mortality is per 1000 live birth, CI: confidence Intervals, Data in bold means *p* < 0.05.

Univariable analyses (Table 3) showed that, increased parity, sex, place of birth, mode of delivery, being born preterm, lower birth weight, and primary diagnosis of congenital anomalies or prematurity were associated with increase in neonatal mortality rate ratios. In contrast, increase in gestational age, increase in neonatal age, antenatal attendance, and primary diagnosis of sepsis, hypothermia, jaundice, glycaemia (hyper or hypo), stable neonates were associated with decrease in neonatal mortality rate ratios. For instance, compared with those born in the hospitals, being born in the health centre and at home were significantly associated with 46% (RR: 1.46, 95% CI 1.22–1.75) and 75% (RR: 1.75, 95% CI 1.51–2.02) increase in neonatal mortality rate ratios, respectively. Similarly, being born preterm (< 37 weeks), having low birth weight (< 2500 g), and having a primary diagnosis of congenital anomalies after birth were significantly associated with 144% (RR: 2.44, 95% CI 2.09–2.84), 143% (RR: 2.43, 95% CI 2.16–2.73), and 69% (RR: 1.69, 95% CI 1.44–1.97) increase in neonatal mortality rate ratios, respectively.

**Table 3:** Univariable and multivariable analyses of factors associated with neonatal mortality (n = 2340).

Variable	Neonatal mortality (n = 2340)	
	CRR (95% CI)	ARR (95% CI)
Time (years)	**0.87 (0.79–0.96)**	1.06 (0.97–1.16)
Age of mother in years	1.01 (0.99–1.01)	–
Gestational age in weeks	**0.91 (0.90–0.92)**	1.00 (0.99–1.01)
Gravida	1.01 (0.98–1.04)	–
Parity	**1.04 (1.00–1.07)**	1.01 (0.98–1.05)
Age of neonate in days	**0.95 (0.93–0.97)**	**0.96 (0.95–0.97)**
Sex		
Male	Ref (1.00)	Ref (1.00)
Female	**1.12 (1.01–1.24)**	1.04 (0.96–1.13)
Place of birth		
Hospital	Ref (1.00)	Ref (1.00)
Health centre	**1.46 (1.22–1.75)**	**1.48 (1.25–1.75)**
Home	**1.75 (1.51–2.02)**	**1.70 (1.46–1.96)**
Mode of delivery		
Spontaneous VD	Ref (1.00)	Ref (1.00)
Caesarean section	**1.40 (1.25–1.58)**	1.51 (0.96–2.36)
Assisted breech VD	**2.45 (2.21–2.74)**	**1.69 (1.47–1.94)**
Vacuum-assisted VD	**2.52 (2.13–2.98)**	**2.39 (1.48–3.86)**
Delivered by		
Midwife	Ref (1.00)	Ref (1.00)
Doctor	**1.46 (1.30–1.64)**	1.09 (0.70–1.68)
Unskilled staff	**1.83 (1.57–2.12)**	0.91 (0.77–1.10)
Maternal HIV status		
Known negative	Ref (1.00)	Ref (1.00)
Known positive	**1.23 (1.09–1.39)**	1.01 (0.91–1.13)
Unknown	1.11 (0.92–1.33)	1.01 (0.85–1.20)
Attended antenatal care		
No	Ref (1.00)	Ref (1.00)
Yes	**0.61 (0.54–0.68)**	**0.76 (0.68–0.85)**
Unknown	**0.31 (0.23–0.44)**	**0.37 (0.27–0.51)**
Preterm birth		
No (≥ 37 weeks)	Ref (1.00)	Ref (1.00)
Yes (< 37 weeks)	**2.44 (2.09–2.84)**	**1.59 (1.36–1.85)**
Low birth weight		
No(≥ 2500 g)	Ref (1.00)	Ref (1.00)
Yes (< 2500 g)	**2.43 (2.16–2.73)**	**1.57 (1.37–1.79)**
Primary diagnosis		
Birth Asphyxia	Ref (1.00)	Ref (1.00)
Congenital anomalies	**1.69 (1.44–1.97)**	**1.59 (1.34–1.88)**
Prematurity	**1.37 (1.23–1.54)**	1.00 (0.80–1.01)
Respiratory distress	0.85 (0.69–1.05)	**0.75 (0.62–0.90)**
Sepsis	**0.45 (0.36–0.56)**	**0.49 (0.40–0.61)**
Hypothermia	**0.37 (0.21–0.66)**	**0.36 (0.22–0.60)**
Jaundice	**0.33 (0.20–0.53)**	**0.41 (0.25–0.67)**
Glycaemia	**0.29 (0.13–0.66)**	**0.35 (0.17–0.74)**
Others	**0.25 (0.16–0.41)**	**0.29 (0.18–0.46)**
Stable neonate	**0.03 (0.01–0.19)**	**0.03 (0.01–0.19)**

*CRR* Crude Rate Ratio, *ARR*: Adjusted Rate Ratio, *CI* confidence interval, *VD* vaginal delivery, *HIV* human immunodeficiency virus. Data in bold means *p* < 0.05.

On the contrary, a 1-day increase in neonatal age, 1-week increase in gestational age, antenatal attendance (Yes), and primary diagnosis of sepsis were significantly associated with 5% (RR: 0.95, 95% CI 0.93–0.97), 9% (RR: 0.91, 95% CI 0.90–0.92), 39% (RR: 0.61, 95% CI 0.54–0.68), and 55% (RR: 0.45, 95% CI 0.36–0.56) reduction in neonatal mortality rate ratios, respectively.

In the multivariable model, being born at the health centre or home, breech delivery, vacuum assisted delivery, being born preterm, having low birth weight, and having a primary diagnosis of congenital anomalies after birth were independently associated with increase in neonatal mortality rate ratios. On the other hand, increase in neonatal age, antenatal attendance and primary diagnoses of respiratory distress syndrome, sepsis, hypothermia, jaundice, glycaemia, stable neonates were also independently associated with decrease in neonatal mortality rate ratios.

For example, being born at home, being born preterm, a primary diagnosis of congenital anomalies, having low birth weight, and being born in the health centre remained independently associated with 70% (ARR: 1.70, 95% CI 1.46–1.96), 59% (ARR: 1.59, 95% CI 1.36–1.85), 59% (ARR: 1.59, 95% CI 1.34–1.88), 57% (ARR: 1.57, 95% CI 1.37–1.79), and 48% (ARR: 1.48, 95% CI 1.25–1.75) increase in neonatal mortality rate ratios, respectively. Conversely, primary diagnosis of hypothermia, antenatal attendance (yes), and one-day increase in neonatal age were independently associated with 64% (ARR: 0.36, 95% CI 0.22–0.60), 24% (ARR: 0.76, 95% CI 0.68–0.85,), and 4% (ARR: 0.96, 95% CI 0.95–0.97) reduction in neonatal mortality rate ratios, respectively.

### Associations with neonatal mortality stratified by gestational age and birth weight

The strength of the association varies by gestational age at birth and birth weight in the univariable stratified analyses (Tables 4 and 5). While sex, antenatal attendance, and low birth weight associations with neonatal mortality rate ratios were stronger for neonates born preterm (< 37 weeks gestation), stronger associations were observed with congenital anomalies, mode of delivery and increase age of neonates for those neonates born at term (≥ 37 weeks gestation). A similar pattern of associations with neonatal mortality rate ratios were observed for birth weight. For instance, 1-day increase in age of neonate, mode of delivery, congenital anomalies, and antenatal attendance were strongly associated with neonatal mortality rate ratios for neonates born with normal birth weight (≥ 2500 g) while only sex and being born preterm were strongly associated with neonatal mortality rate ratios for neonates with low birth weight (< 2500 g).

**Table 4:** Univariable and multivariable analyses of factors associated with neonatal mortality stratified by gestational age (Preterm vs. Term babies).

Variable	Neonatal mortality (n = 2340)			
	Gestation < 37 weeks (n = 1612)		Gestation ≥ 37 weeks (n = 728)	
	CRR (95% CI)	ARR (95% CI)	CRR (95% CI)	ARR (95% CI)
Time (years)	**1.16 (1.05–1.28)**	**1.19 (1.08–1.30)**	**0.49 (0.37–0.65)**	**0.66 (0.50–0.88)**
Age of mother in years	**1.02 (1.01–1.03)**	1.00 (0.99–1.01)	0.99 (0.96–1.01)	–
Gravida	**1.04 (1.01–1.07)**	0.97 (0.91–1.03)	0.93 (0.83–1.03)	–
Parity	**1.04 (1.01–1.07)**	1.04 (0.97–1.11)	0.98 (0.88–1.09)	–
Age of neonate in days	**0.96 (0.94–0.98)**	**0.97 (0.96–0.98)**	**0.88 (0.82–0.95)**	**0.90 (0.85–0.96)**
Sex				
Male	Ref (1.00)	Ref (1.00)	Ref (1.00)	Ref (1.00)
Female	**1.14 (1.03–1.26)**	1.07 (0.98–1.17)	0.88 (0.65–1.17)	–
Place of birth				
Hospital	Ref (1.00)	Ref (1.00)	Ref (1.00)	Ref (1.00)
Health centre	1.18 (0.97–1.44)	**1.39 (1.15–1.67)**	**2.94 (1.97–4.39)**	**1.51 (1.04–2.18)**
Home	**1.74 (1.56–1.96)**	**1.74 (1.49–2.01)**	0.47 (0.12–1.77)	0.54 (0.15–1.95)
Mode of delivery				
Spontaneous VD	Ref (1.00)	Ref (1.00)	Ref (1.00)	Ref (1.00)
Caesarean section	**1.47 (1.31–1.65)**	1.35 (0.85–2.16)	**1.73 (1.26–2.36)**	1.68 (0.75–3.77)
Assisted breech VD	**2.07 (1.88–2.29)**	**1.65 (1.45–1.88)**	**3.52 (1.68–7.36)**	1.41 (0.79–2.51)
Vacuum VD	**1.98 (1.56–2.51)**	**2.06 (1.19–3.59)**	**5.86 (4.90–7.01)**	**3.54 (2.35–5.36)**
Delivered by				
Midwife	Ref (1.00)	Ref (1.00)	Ref (1.00)	Ref (1.00)
Doctor	**1.53 (1.37–1.71)**	1.11 (0.71–1.69)	**1.72 (1.27–2.32)**	1.32 (0.62–2.80)
Unskilled staff	**1.85 (1.64–2.08)**	0.94 (0.77–1.10)	0.51 (0.13–1.92)	0.95 (0.56–1.34)
Maternal HIV status				
Known negative	Ref (1.00)	Ref (1.00)	Ref (1.00)	Ref (1.00)
Known positive	**1.21 (1.08–1.36)**	1.01 (0.91–1.12)	0.96 (0.63–1.47)	–
Unknown	1.10 (0.92–1.31)	1.02 (0.86–1.22)	0.79 (0.39–1.59)	–
Attended antenatal care				
No	Ref (1.00)	Ref (1.00)	Ref (1.00)	Ref (1.00)
Yes	**0.69 (0.62–0.77)**	**0.77 (0.68–0.85)**	**0.49 (0.33–0.72)**	**0.68 (0.48–0.96)**
Unknown	**0.41 (0.29–0.56)**	**0.41 (0.28–0.53)**	**0.05 (0.01–0.37)**	**0.11 (0.02–0.74)**
Low birth weight				
No(≥ 2500 g)	Ref (1.00)	Ref (1.00)	Ref (1.00)	Ref (1.00)
Yes (< 2500 g)	**3.86 (3.07–4.85)**	**1.73 (1.62–2.10)**	1.10 (0.73–1.65)	**–**
Primary diagnosis				
Birth Asphyxia	Ref (1.00)	Ref (1.00)	Ref (1.00)	Ref (1.00)
Congenital anomalies	**1.40 (1.17–1.67)**	**1.58 (1.34–1.87)**	**2.15 (1.60–2.88)**	**2.19 (1.55–3.11)**
Prematurity	1.12 (0.99–1.27)	0.95 (0.84–1.07)	0.86 (0.45–1.65)	0.84 (0.47–1.53)
Respiratory distress	0.89 (0.72–1.10)	**0.77 (0.65–0.93)**	**0.45 (0.24–0.84)**	**0.54 (0.31–0.98)**
Sepsis	**0.46 (0.36–0.59)**	**0.51 (0.41–0.66)**	**0.34 (0.21–0.56)**	**0.44 (0.27–0.71)**
Hypothermia	**0.45 (0.26–0.78)**	**0.38 (0.23–0.67)**	**0.01 (0.02–0.08)**	**0.04 (0.02–0.07)**
Jaundice	**0.39 (0.23–0.67)**	**0.41 (0.28–0.83)**	**0.20 (0.07–0.61)**	0.33 (0.11–1.01)
Glycaemia	**0.38 (0.16–0.92)**	**0.44 (0.20–0.95)**	0.15 (0.02–1.05)	**0.21 (0.04–0.99)**
Others	**0.32 (0.18–0.56)**	**0.39 (0.23–0.68)**	**0.21 (0.09–0.47)**	**0.18 (0.08–0.37)**
Stable neonate	**0.04 (0.01–0.26)**	**0.05 (0.01–0.32)**	**0.02 (0.01–0.09)**	**0.02 (0.01–0.07)**

*CRR* Crude Rate Ratio, *ARR*: Adjusted Rate Ratio, *CI* confidence interval, *VD* vaginal delivery, *HIV* human immunodeficiency virus. Data in bold means *p* < 0.05.

**Table 5:** Univariable and multivariable analyses of factors associated with neonatal mortality stratified by birth weight (Low birthweight versus normal birthweight).

Variable	Neonatal mortality (n = 2340)			
	Birthweight < 2500 g (n = 1192)		Birthweight ≥ 2500 g (n = 1148)	
	CRR (95% CI)	ARR (95% CI)	CRR (95% CI)	ARR (95% CI)
Time (years)	**1.04 (1.01–1.15)**	**1.14 (1.04–1.26)**	**0.70 (0.57–0.87)**	0.83 (0.68–1.01)
Age of mother in years	**1.02 (1.01–1.03)**	**1.02 (1.01–1.04)**	0.98 (0.96–1.00)	**–**
Gravida	**1.05 (1.02–1.08)**	1.02 (0.97–1.09)	0.93 (0.85–1.01)	–
Parity	**1.05 (1.02–1.08)**	0.96 (0.91–1.03)	1.00 (0.91–1.10)	–
Age of neonate in days	**0.95 (0.94–0.97)**	**0.97 (0.95–0.98)**	**0.93 (0.88–0.97)**	**0.94 (0.90–0.97)**
Sex				
Male	Ref (1.00)	Ref (1.00)	Ref (1.00)	Ref (1.00)
Female	**1.12 (1.01–1.24)**	**1.14 (1.04–1.24)**	0.91 (0.73–1.12)	–
Place of birth				
Hospital	Ref (1.0 0)	Ref (1.00)	Ref (1.00)	Ref (1.00)
Health centre	**1.99 (1.40–2.80)**	**1.68 (1.24–2.27)**	1.19 (0.98–1.45)	**1.36 (1.12–1.64)**
Home	1.01 (0.52–1.95)	1.25 (0.66–2.36)	**1.58 (1.41–1.76)**	**1.70 (1.47–1.98)**
Mode of delivery				
Spontaneous VD	Ref (1.00)	Ref (1.00)	Ref (1.00)	Ref (1.00)
Caesarean section	**1.61 (1.27–2.04)**	**2.74 (1.61–4.62)**	**1.57 (1.42–1.74)**	1.05 (0.70–1.56)
Assisted breech VD	**3.33 (2.06–5.39)**	**1.89 (1.27–2.82)**	**1.84 (1.68–2.02)**	**1.58 (1.41–1.79)**
Vacuum-assisted VD	**4.55 (3.62–5.71)**	**3.59 (2.39–5.38)**	**1.94 (1.83–2.06)**	1.16 (0.74–1.82)
Delivered by				
Midwife	Ref (1.00)	Ref (1.00)	Ref (1.00)	Ref (1.00)
Doctor	**1.65 (1.32–2.07)**	0.75 (0.46–1.22)	**1.62 (1.47–1.79)**	1.41 (0.96–2.08)
Unskilled staff	1.09 (0.56–2.12)	0.91 (0.86–1.19)	**1.67 (1.49–1.88)**	0.92 (0.78–1.08)
Maternal HIV status				
Known negative	Ref (1.00)	Ref (1.00)	Ref (1.00)	Ref (1.00)
Known positive	0.98 (0.72–1.34)	–	**1.17 (1.05–1.32)**	1.01 (0.91–1.13)
Unknown	1.01 (0.66–1.56)	–	1.04 (0.87–1.25)	0.93 (0.78–1.11)
Attended antenatal care				
No	Ref (1.00)	Ref (1.00)	Ref (1.00)	Ref (1.00)
Yes	**0.55 (0.41–0.73)**	**0.63 (0.48–0.81)**	**0.72 (0.65–0.81)**	**0.77 (0.69–0.86)**
Unknown	**0.14 (0.06–0.34)**	**0.17 (0.07–0.40)**	**0.50 (0.36–0.68)**	**0.47 (0.34–0.63)**
Preterm				
No (≥ 37 weeks)	Ref (1.00)	Ref (1.00)	Ref (1.00)	Ref (1.00)
Yes (< 37 weeks)	**2.71 (1.83–3.99)**	**2.23 (1.61–3.28)**	**1.37 (1.11–1.70)**	**1.30 (1.07–1.56)**
Primary diagnosis				
Birth Asphyxia	Ref (1.00)	Ref (1.00)	Ref (1.00)	Ref (1.00)
Congenital anomalies	**1.57 (1.21–2.04)**	**1.86 (1.39–2.47)**	**1.50 (1.23–1.82)**	**1.77 (1.44–2.16)**
Prematurity	0.50 (0.22–1.09)	0.56 (0.24–1.28)	1.10 (0.93–1.29)	1.14 (0.98–1.31)
Respiratory distress	**0.42 (0.27–0.67)**	**0.46 (0.30–0.73)**	1.03 (0.82–1.30)	1.07 (0.87–1.32)
Sepsis	**0.33 (0.23–0.45)**	**0.36 (0.26–0.50)**	**0.65 (0.48–0.87)**	0.79 (0.60–1.04)
Hypothermia	**0.02 (0.01–0.03)**	**0.02 (0.01–0.07)**	0.68 (0.41–1.13)	0.72 (0.45–1.16)
Jaundice	**0.39 (0.22–0.70)**	**0.51 (0.29–0.90)**	**0.21 (0.08–0.53)**	**0.33 (0.14–0.83)**
Glycaemia	**0.09 (0.01–0.59)**	**0.11 (0.02–0.67)**	0.71 (0.33–1.53)	0.85 (0.44–1.65)
Others	**0.18 (0.09–0.34)**	**0.17 (0.09–0.32)**	0.65 (0.35–1.19)	0.86 (0.48–1.56)
Stable neonate	**0.04 (0.01–0.27)**	**0.03 (0.01–0.19)**	**0.05 (0.02–0.09)**	**0.05 (0.03–0.09)**

*CRR* Crude Rate Ratio, *ARR*: Adjusted Rate Ratio, *CI* confidence interval, *VD* vaginal delivery, *HIV* human immunodeficiency virus. Data in bold means *p* < 0.05.

In the multivariable analyses (Table 4), several factors including age of neonate, antenatal attendance, vacuum delivery, being delivered at health centre, congenital anomalies, respiratory distress, sepsis, hypothermia were independently associated with neonatal mortality rate ratios in both preterm and term neonates. Interestingly, being born at health centre and home, breech-assisted and vacuum delivery, and low birth weight were independently associated with increase in neonatal mortality rate ratios in preterm neonates. In Table 5, similar several common factors including age of neonate, antenatal attendance, breech delivery, being deliver at health centre, congenital anomalies, jaundice, and being born preterm were independently associated with neonatal mortality rate ratios in both normal weight and low birth weight neonates. However, caesarean-section, breech-assisted and vacuum-assisted delivery as well as respiratory distress syndrome, sepsis, hypothermia, and glycaemia were independently associated with neonatal mortality rate ratios in neonates with low birth weight.

## Discussion

In this study, we examined the trends and determinants of neonatal mortality of neonates admitted to the women and new-born tertiary hospital in Lusaka, Zambia between 2018 and 2019. Overall, we found that 40% (402/100,000 live births) of neonates admitted to the NICU died over the 2-years period with a 6% (56/100,000 live births) slight decline from 2018 to 2019. Being born at health centre and home, breech and vacuum modes of delivery, preterm birth, low birth weight and congenital anomalies were independently associated with increase in neonatal mortality. On the contrary, increase in neonatal age, antenatal attendance and primary diagnosis of respiratory distress, sepsis, hypothermia, jaundice, glycaemia, and stable neonates were independently associated with decrease in neonatal mortality. These associations were generally stronger for neonates born preterm or with low birthweight compared to neonates born at term or with normal birthweight. These findings are important and seem to suggest that efforts at increasing skilled birth at health facility or home, early interventions in reducing pregnancy/birth complications, and other adverse birth outcomes are still vital in reducing neonatal mortality rates.

Contrary to our expectations, we found a higher cumulative neonatal mortality over the 2-years period, with a gradual decrease in trend from 2018 to 2019 in a higher level of referral system where high quality of care and expertise are expected. These rates are much higher than the general national neonatal mortality rates of 32/1000 live births in 2018 and 33/1000 in 2019^28,29^. The UTH-NICU in Lusaka receives major referrals across the country and from surrounding communities and it is possible that most neonates referred to the facility were in critical condition and therefore were subject to low survival rates. In addition, there may be delays in or inappropriate obstetric referrals from lower-level health facilities suggesting that late arrivals for life-saving interventions, overwhelmed referral facilities or lack of timely comprehensive obstetric care may have compromised quality of care and consequently reduced neonatal survival rates. These may have accounted for the high neonatal mortality rates observed in this study. Considering that Lusaka alone contributes 19% of the country’s total population and that Zambia is one of the countries with higher urban than rural neonatal mortality rates^4^, efforts to improve neonatal health outcomes in Lusaka may significantly contribute to the reduction of neonatal mortality in the country.

We found that several factors were associated with increase in neonatal mortality. For example, being born at home, or health centre compared with hospital, breech and vacuum delivery compared with spontaneous vaginal were associated with 70%, 48%, 69%, and 39%, increase in neonatal mortality rates, respectively. Similar to our findings, home and health centre delivery were found to be significant risk factors for neonatal mortality in Africa including Zambia^8^. Lower maternal education, inability to pay for cost of care at the hospital, health care worker attitudes, religious beliefs, long travel distance and lack of husband approval were some of the factors attributable to home delivery^8^. Previous studies in Ethiopia^30–32^, Benin^16^, and Eretria^18^ also found breech and vacuum deliveries to be significant risk factors for neonatal mortality in neonates referred to NICUs. Continual education of health care workers especially nurses and midwives in emergency obstetric care, timely risks assessment and timely referral of high-risk expectant mothers, improved breech and vacuum deliveries procedure and management, and neonatal resuscitation may contribute to the reduction of neonatal mortality^3,14^. In addition, appropriate education of expectant mothers empathizing regular antenatal visits, early birth preparedness involving their husbands, and health facility deliveries may assist mothers to have safe deliveries and reduce neonatal mortality^3,14^. Improvement in community and health centre level referral systems to reduce transport delays and establishment of functional mothers’ shelter near health facilities may assist those far away to have easy and early access to referral sites for appropriate interventions that may contribute to a reduction in neonatal mortality^3,14^.

In addition, we found congenital anomalies to be associated with 59% increased risk of neonatal mortality in our study with stronger effect sizes observed in preterm and low birthweight neonates. The global incidence of congenital anomalies is about 3–4% in all births. Globally, congenital anomalies are the fourth leading cause of neonatal mortality with an annual death of 240,000 of which 90% with serious disorders are from low-and-middle-income countries^33^. Our findings are similar to previous studies in Nigeria^34^, Ghana^35^, and South Africa^36^ where congenital anomalies was found to have accounted for 10.4–33.5% of neonatal mortality of neonates admitted to NICU. Measures to promote primary prevention and improve the health of neonates with congenital disorders via improved surveillance, building capacity and increase expertise on prevention and care, increasing awareness on newborn screenings, strengthening research on major birth defects, and supporting affected families may help reduce the burden of congenital anomalies^33^.

Predictably, we found that antenatal attendance was associated with 24% decrease in neonatal mortality. Similarly, previous survival analysis of risk factors of neonatal mortality in low-middle-income countries indicated that one antenatal attendance lowers the risk of neonatal mortality by 26% and this increased to 51% lower risk if the antenatal visits are up to four^37^. A recent meta-analysis also highlighted that less than four antenatal visits increase the risk of neonatal mortality by 1.76 times compared to those that had four antenatal visits^38^. Adequate antenatal visits are important to help in early detection and appropriate management of birth-related problems to reduce pregnancy/birth complication and the risk of neonatal death.

Interestingly, we found that primary diagnosis of glycaemia (hypoglycaemia and hyperglycaemia), hypothermia, jaundice, sepsis, and respiratory distress syndrome were associated with 65%, 64%, 59%, 51%, and 25% decrease in neonatal morality, respectively. These are important findings and highlights that measures implemented by the Zambia government and its partners to address common preventable neonatal death in the health facilities appears to be yielding the expected outcomes. Several strategic policy and implementation-related decisions were made between 2000 and 2017 in areas of capacity building of health care workers, healthcare infrastructure improvement, increase availability of skilled birth attendance, and community outreach programs to encourage facility level delivery. Implementation of key strategic programs such as maternal and perinatal death review, essential newborn care, Kangaroo mother care, helping babies breathe, Safe Motherhood practices, and interventions using the EmONC recommendations may have contributed significantly to the reduction of preventable neonatal death observed in this study^3,39^.

In general, associations with neonatal mortality were stronger for preterm (< 37 weeks gestation) than for term neonates (≥ 37 weeks gestation) and were also stronger for low birthweight (< 2500 g) compared to normal birthweight (≥ 2500 g) neonates. The finding of stronger associations for preterm is consistent with a meta-analysis which found that preterm birth consistently accounted for 5.7–7.1 times higher neonatal mortality rates than term birth^40^. Similarly, the same meta-analysis found low birth weight to have consistently accounted for 9.9–15.5 times higher neonatal mortality rates compared to normal birthweight^40^. This implies that interventions targeting improve care for preterm and low birthweight neonates may reduce neonatal mortality.

The findings of this study should be interpreted taking into consideration its’ limitations. We could not retrieve all case files of neonates admitted at the NICU and amongst those retrieved, many had inadequate information of maternal and perinatal characteristics. Those neonates who died on arrival or who were not registered in the NICU register were not captured. Selection bias may have occurred as not all neonate admitted were included. When we compared this study participants to those not included but have limited information, there were no differences in gestational age, age of neonates on admission, sex, place of birth and birthweight. We further examined whether gestational age, age of neonate on admission, sex, place birth and birthweight confounded our relationships but this was not the case. This supports the view that population differences may affect prevalence estimates but are not likely to substantially affect exposure-outcome relationships^41^. Nonetheless, considering that this is a hospital-based retrospective study, the findings cannot be generalizable to the general neonatal population. Despite this, the study highlights the trends and determinants of neonatal morality in a major referral centre in the country which is important for policy interventions.

## Conclusion

Neonatal mortality in a major referral centre in Zambia highlights a slight reduction between 2018 and 2019 implying that current efforts towards achieving the neonatal mortality SDG goal is gradually being achieved. The findings of our study indicate that some of the factors associated with neonatal mortality especially in the NICU are being successfully implemented by the government of Zambia. It is therefore important to continue with the improvement in prenatal and intrapartum care, obstetric emergency services, and postnatal care geared towards the reduction of neonatal mortality in Zambia. It is also important to continue to monitor the trends and cause-specific neonatal mortality at all levels especially in referral hospitals as well as rural urban disparities to support improve quality of care and key decision-making. Our study findings provide important information and pointers to guide Government and partners in identifying and prioritizing key neonatal mortality problems in their health policy development and interventions.

## Data Availability

The datasets used in this current study is available from the corresponding author on reasonable request.

## Abbreviations

AIC: Akaike information criterion
EmONC: Emergency obstetric and neonatal care
FETP: Field Epidemiology Training Program
HMIS: Health management information system
MOH: Ministry of Health
NICU: Neonatal intensive care unit
NMR: Neonatal mortality rate
SDGs: Sustainable Developmental Goals
UTH: University Teaching Hospital
